# Influence of planting yellowhorn (*Xanthoceras sorbifolium* Bunge) on the bacterial and fungal diversity of fly ash

**DOI:** 10.7717/peerj.14015

**Published:** 2022-09-23

**Authors:** Zehui Liu, Jianguo Zhao, Jinxian Huo, Hongfang Ma, Zhiwen Chen

**Affiliations:** 1 Institute of Carbon Materials Science, School of Chemistry and Chemical Engineering, Shanxi Datong University, Datong, Shanxi, China; 2 Hainan Yazhou Bay Seed Laboratory, Sanya, Hainan, China

**Keywords:** Fly ash, Microbial diversity, Physicochemical properties, Phytoremediation, Yellowhorn

## Abstract

Phytoremediation is a low-cost solution to fly ash pollution and the rhizosphere interactions between plant roots and the fly ash microbiome were important for the phytoremediation. To analyze the dynamic changes of the rhizosphere microbiome during yellowhorn cultivation in fly ash, the bacterial 16S rRNA gene V3–V4 region and the fungal ITS region of the rhizosphere microbiome were sequenced using Illumina MiSeq technology. The changes in fly ash physicochemical properties and the heavy metal content of different yellowhorn tissues were also analyzed. The results showed that both the bacterial and fungal communities were noticeably different after yellowhorn cultivation compared with the control sample. *Proteobacteria* and *Acidobacteria* levels increased (*p* < 0.05) and *Firmicutes* and *Actinobacteria* decreased (*p* < 0.05) in the bacterial community after yellowhorn cultivation. In the fungal community, *Ascomycota* and *Mortierellomycota* decreased (*p* < 0.05), while *Chytridiomycota* increased (*p* < 0.05). The levels of four heavy metals (Cr, Cd, Hg, Pb and As) decreased in the fly ash after yellowhorn cultivation. These metals were absorbed by the yellowhorn plants and accumulated in the fibrous root, taproot, stem and leaf tissues of these plants. Accordingly, the abundance of bacteria that could solubilize heavy metals increased (*p* < 0.05). In summary, the cultivation of yellowhorn affected the composition of the rhizosphere microbial communities in fly ash, which is of great significance for the biological remediation of fly ash.

## Introduction

Yellowhorn (*Xanthoceras sorbifolium* Bunge) is a type of oil tree that has developed roots able to resist adverse growing conditions such as cold, drought, and high salinity levels (Lang et al., 2020; Liu et al., 2017; Shen et al., 2019). Yellowhorn is widely distributed throughout China and belongs to the family Sapindaceae and genus Xanthoceras. It is capable of growing in a variety of soil types (Zang et al., 2021). Fly ash is a type of solid waste produced from coal-fired power plants. Thermal power generation, which produces a large mass of fly ash after coal combustion, is the main source of energy in China (Ding et al., 2017). One tonne of coal combustion can produce about 250–300 kg of fly ash, mainly composed of Al_2_O_3_, SiO_2_, Fe_2_O_3_, CaO, TiO_2_, MgO, Na_2_O, and heavy metals such as As, Cr, Cd, Pb, Hg, and Se (Blissett & Rowson, 2012; Pandey & Singh, 2014). China currently consumes up to 1.8 billion tonnes of coal for thermal power generation every year with 450 million tonnes of fly ash produced during the process (Han et al., 2021). In order to help improve soil and water pollution, around 70% of the fly ash produced in China is currently being utilized, primarily in cement production for building materials (Ding et al., 2017; Jala & Goyal, 2006; Wan, Xu & Wang, 2015; Zhang, Wen & Li, 2021; Zhao et al., 2021).

Fly ash can cause serious air and water pollution (Tiwari, Kumari & Singh, 2008) and has encroached on both forests and agricultural land (Kalra et al., 1998). Fully utilizing all fly ash produced would help combat these problems. Phytoremediation, which uses plants to extract and remove elemental pollutants in soil, has emerged as a primary solution to fly ash pollution because of its low cost (Garbisu & Alkorta, 2001; Yan et al., 2020). Research supports this solution. For example, planting *Astragalus sinicus*, *Spinacea oleracea* and *Lolium perenne* crops was found to reduce the salt content of greenhouse soil (Cai et al., 2022). *Cannabis sativa* is also capable of capturing metals from the environment through phytoremediation (Placido & Lee, 2022). One study found that the polycyclic aromatic hydrocarbons in fly ash were reduced by 50% after 3 years of phytoremediation with willows (Kosnar, Mercl & Tlustos, 2020). Naturally growing weeds have also been used on fly ash-amended soil for healthy soil restoration (Panda, Mandal & Barik, 2020) and other plants have also been able to remove the contaminating components of fly ash when grown in coal fly ash (Kisku et al., 2018). All these studies show that phytoremediation is an ideal plant-based approach to comprehensively utilize fly ash. However, the interaction mechanism between plant roots and fly ash soil is still not well understood.

The rhizosphere is a unique region where interactions occur between plant roots and the soil microbiome (Mendes, Garbeva & Raaijmakers, 2013). Rhizosphere microorganisms can regulate substrate and energy transformations to improve soil fertility (Breidenbach, Pump & Dumont, 2015). High-throughput DNA sequencing technology has enabled the comprehensive analysis of rhizosphere microbial community diversity (Bulgarelli et al., 2012; Edwards et al., 2015; Lundberg et al., 2012; Peiffer et al., 2013) and researchers have found that there are significant differences in the community diversity of rhizosphere microbes between different plant species (Sugiyama et al., 2014; Zimudzi et al., 2018). In addition, a study of plant-microbial interactions found that during the plant growth process, plant root exudates promote the growth and activity of the rhizosphere microbial community (Saravanan et al., 2020).

Because of yellowhorn’s ability to grow in a variety of soil types and because it has developed roots able to withstand adverse growing conditions, it is an ideal plant to cultivate in fly ash for soil remediation. To test this hypothesis, and to better understand the changes to the microbial diversity in the rhizosphere after yellowhorn cultivation in fly ash, the bacterial 16S rRNA genes and the fungal ITS region of the rhizosphere microbiome were sequenced using the Illumina HiSeq amplicon sequencing method after the cultivation of yellowhorn compared with a pre-planting sample.

## Materials and Methods

### Experimental design

This experiment was carried out in the Institute of Carbon Materials Science of Shanxi Datong University, and the fly ash samples were collected from the Datong Thermal Power Plant. Yellowhorn seeds of an identical size were germinated in potting fly ash in a growth chamber, and the resulting seedlings were maintained in a controlled environment at 28 °C day/20 °C night, with a 16-h light/8-h dark photoperiod. The fly ash samples without yellowhorn seedlings were defined as the control check (or CK) group, while the fly ash samples cultivated by yellowhorn were defined as the seedlings test (or S) group.

### Sampling of rhizosphere soil and yellowhorn tissues

Bulk fly ash samples and rhizosphere fly ash samples were collected on June 15, 2020 from both the control (CK) and one-year-old yellowhorn sampling (S) groups. A total of 30 groups of parallel experiments were conducted for both the CK and S groups. Three biological samples were performed with each replication mixing five random rhizosphere fly ash samples. First, the roots in the rhizosphere fly ash were removed. The physical and chemical properties of the fly ash were determined using a sample with a particle size less than 2 mm. Rhizosphere fly ash samples were frozen with liquid nitrogen and stored in a refrigerator at −80 °C prior to the extraction of soil DNA for amplicon sequencing. The root, stem and leaf samples of the yellowhorn were dried in the oven at 70 °C for 24 h and then used to measure heavy metal content with three biological replications (Davidson, 2013).

### Determining the heavy metal content of different yellowhorn tissues

The heavy metal content, specifically the levels of Pb, Hg, Cr, Cd and As, in the different yellowhorn tissues were determined by microwave digestion coupled with the plasma-mass spectrometry method (ICAP6300) in the lab of Prof. Guanghui Xie at China Agricultural University. First, microwave-assisted digestion was applied to digest the yellowhorn root, stem and leaf samples. For each plant sample, 7 ml nitric acid (HNO_3_ 63%) and 2 ml hydrogen peroxide (H_2_O_2_ 72%) were used for the digestion of plant tissues with a microwave digester (Milestone ETHOS ONE, Leutkirch im Allgau, Germany) for 2 h. After digestion, the sample was heated on a hot plate at 135 °C for 2 h. It was then cooled to room temperature using double distilled water to achieve a final volume of 50 ml. Finally, the samples were filtered using a 0.22 μm membrane filter and analyzed using inductively coupled plasma mass spectrometry (ICP-MS; ThermosXSERIES2).

### Determining the physical and chemical properties of the fly ash

The pH of the fly ash was measured using a calibrated pH meter (Thermo Orion, Waltham, MA, USA) and the morphology of the fly ash was characterized using a scanning electron microscope (TESCAN MAIA3LMH, Czech Republic). The physical and chemical properties of the fly ash, including the heavy metal content (Pb, Hg, Cr, Cd and As), were also determined. Heavy metal elements in the fly ash were measured by microwave digestion coupled with plasma mass spectrometry (ICAP6300; Mao et al., 2017).

### Extracting fly ash DNA and sequencing with Illumina HiSeq 2500

Total DNA was extracted from each fly ash sample (0.3 g), including three biological replicates from both the CK and S groups using the NucleoSpin 96 Soil kit (MACHEREY-NAGEL, Düren, Germany); 30 ng of fly ash DNA was used for the subsequent PCR analysis. Primers 338F (5′-ACTCCTACGGGAGGCAGCAG-3′) and 806R (5′-GGACTACHVGGGTWTCTAAT-3′) were used to amplify the prokaryotic 16S rRNA gene V3–V4 region. Primers ITS1-F (5′-CTTGGTCATTTAGAGGAAGTAA-3′) and ITS2-R (5′-TGCGTTCTTCATCGATGC-3′) were used to amplify the fungal internal transcribed spacer (ITS) regions ITS1–ITS2 of the ribosomal RNA gene. PCR thermocycling was performed starting with 5 min at 95 °C, followed by 25 cycles of: 30 s at 95 °C, 30 s at 50 °C, and 40 s at 72 °C, with a final extension at 72 °C for 7 min and a hold temperature of 16 °C. The PCR products were checked using 1% agarose gel electrophoresis and recovered using the Agarose Gel Extraction kit (Thermo Scientific GeneJET, Waltham, MA, USA). Amplicon library preparation and 150-bp paired-end DNA sequencing, using Illumina HiSeq 2500, were performed at Beijing Biomarker Technologies Co, LTD (Beijing, China). Three biological replicates were performed for both the CK and S groups.

### Quality screening of sequencing data

Raw bacterial and fungal sequence reads were evaluated using two quality control tools, the Trimmomatic (Bolger, Lohse & Usadel, 2014) and the FilterReads module in Kmernator (https://github.com/JGI-Bioinformatics/Kmernator). All adapters, low quality or “N” bases, and reads shorter than 50 bp were removed from the data. Then, the FLASH v1.2.11 (Magoc & Salzberg, 2011) software was used to merge the paired-end reads with a minimum overlap length of 50 bp. The raw sequencing data has been submitted to the Genome Sequence Archive (http://gsa.big.ac.cn/) under accession number CRA005791.

### Species annotation and taxonomic analysis

Clean tags with at least 97% similarity were clustered into Operational Taxonomic Units (OTUs) using USEARCH version 10.0 (Edgar, 2013) and filtered using the 0.005% OTU abundance filtering approach (Bokulich et al., 2013). The SILVA database (version 138; http://www.arb-silva.de) and the UNITE database (version 7.2; https://unite.ut.ee) were used to identify the bacterial and fungal OTU representative sequences, respectively, and the RDP Classifier algorithm was used with an 0.8 confidence threshold (Abarenkov et al., 2010; Quast et al., 2013; Wang et al., 2007). Singleton OTUs, those containing only one sequence, were removed from all samples.

High quality OTU sequences from both the bacterial and fungal groups were aligned to the microbial reference database (Release132, http://www.arb-silva.de and Release 8.0, https://unite.ut.ee/) to annotate the corresponding species classification information for each OTU, including phylum, class, order, family, genus and species. Then, the QIIME 2 software was used to generate a species-level abundance table (Caporaso et al., 2010b; Estaki et al., 2020; Lawley & Tannock, 2017), and the community structures were drawn at the taxonomic level using the R language (version 4.0.2; R Core Team, 2021). The bacterial sequences were aligned and a neighbor-joining evolutionary tree was produced using the PyNAST software (version 1.2.2, http://biocore.github.io/pynast/; Caporaso et al., 2010a). The fungal sequences were then aligned and a neighbor-joining tree was constructed using the ClustalW2 software (http://www.ebi.ac.uk/Tools/msa/clustalw2/; Larkin et al., 2007).

### Diversity analysis

The alpha diversity index (Chao1 indexes, Ace indexes, Shannon indices and Simpson indexes) of the samples was evaluated using the Mothur v.1.30 software (Grice et al., 2009). A beta diversity analysis was performed using the QIIME 2 software (Jiang & Takacs-Vesbach, 2017; Jiang et al., 2022). The dataset normalization for the alpha and beta diversity analyses was completed using a rarefaction curve analysis to equal depth. A principal component analysis (PCA), ANOSIM similarity analysis, and Linear discriminant analysis Effect Size (LEfSe) (Segata et al., 2011), were performed using the R language (version 4.0.2; R Core Team, 2021) and SPSS (version 19.0) was used to perform the significance analysis. The LefSe analysis was used to screen the biomarker and compare the q values to identify the significance of differences between the CK and S groups at each classification level (Segata et al., 2011).

## Results

### Particle size distribution of fly ash under scanning electron microscope (SEM)

**Figure 1:**
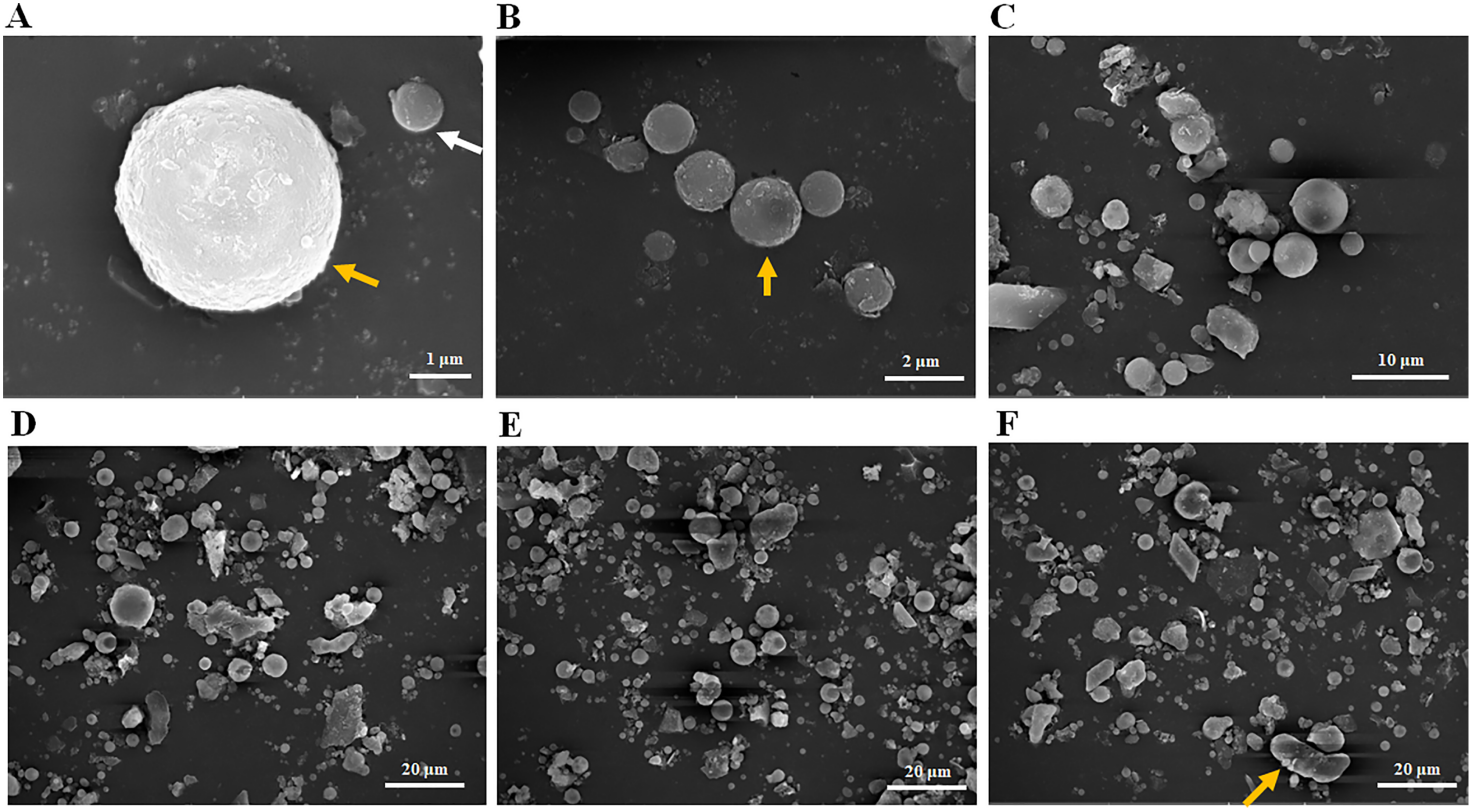
Particle size distribution of fly ash under scanning electron microscope (SEM). (A) Fly ash particles under 1 μm field of vision, (B) fly ash particles under 2 μm field of vision, (C) fly ash particles under 10 μm field of vision, (D) fly ash particles under 20 μm field of vision, (E) fly ash particles under 20 μm field of vision, (F) fly ash particles under 20 μm field of vision.

Scanning electron microscopy was used to observe the particle size distribution of the fly ash used in this experiment. Most fly ash particles with 1 μm (Fig. 1A, white arrow), 2 μm (Fig. 1B, yellow arrow) or 5 μm in size (Fig. 1A, yellow arrow) observed were spherical in shape (Figs. 1A–1C). When a 20 μm field of vision was used (Figs. 1D–1F), most fly ash particles were less than 20 μm in size with very few reaching 20 μm (Fig. 1F, yellow arrow) and most were spherical, but some rod and lamellar structures were also identified (Figs. 1D–1F). In summary, the size distribution of the fly ash particles in this experiment ranged from 1–20 μm (Fig. 1), and most fly ash particles identified were spherical in shape. In addition, the average pH value of the fly ash was 8.32 with 10 repeated trials, classifying it as mildly alkaline ash.

### OTU analyses of bacterial 16S rRNA genes and fungal ITS sequences

A total of 957,118 bacterial 16S rRNA raw sequences (averaging 79,760 and ranging from 79,587 to 80,015 reads per sample; Table S1) and 959,918 fungal ITS raw sequences (averaging 79,993 and ranging from 79,817 to 80,101 reads per sample; Table S2) were generated. After removing the short sequences, adapters, and low-quality sequences, 928,086 16S rRNA high-quality sequences (averaging 77,336 and ranging from 77,049 to 77,611 reads per sample; Table S1) and 899,392 ITS high-quality sequences (averaging 74,949 and ranging from 72,409 to 77,101 reads per sample; Table S2) were obtained for the subsequent analysis. The average lengths of the high-quality bacterial 16S rRNA sequences were between 417 and 419 bp (Table S1), and the high-quality fungal ITS sequences averaged between 234 and 244 bp in size (Table S2). The Shannon-Wiener curve and the species accumulation curve showed that the number of high-quality sequences obtained was sufficient to reflect the microbial diversity and species abundance in the samples (Figs. S1A, S1B). The results of the rarefaction curve analysis showed that there were between 1,091 and 1,642 bacterial OTUs and between 147 and 265 fungal OTUs (Figs. S1C, S1D). The bacterial sequences were clustered into 2,740 OTUs, and the fungal sequences were clustered into 611 OTUs when using a 97% sequence similarity level (Figs. S1E, S1F). These results showed that the bacterial diversity was significantly higher than the fungal.

### Change in rhizosphere microbial community diversity in fly ash after yellowhorn cultivation

**Figure 2:**
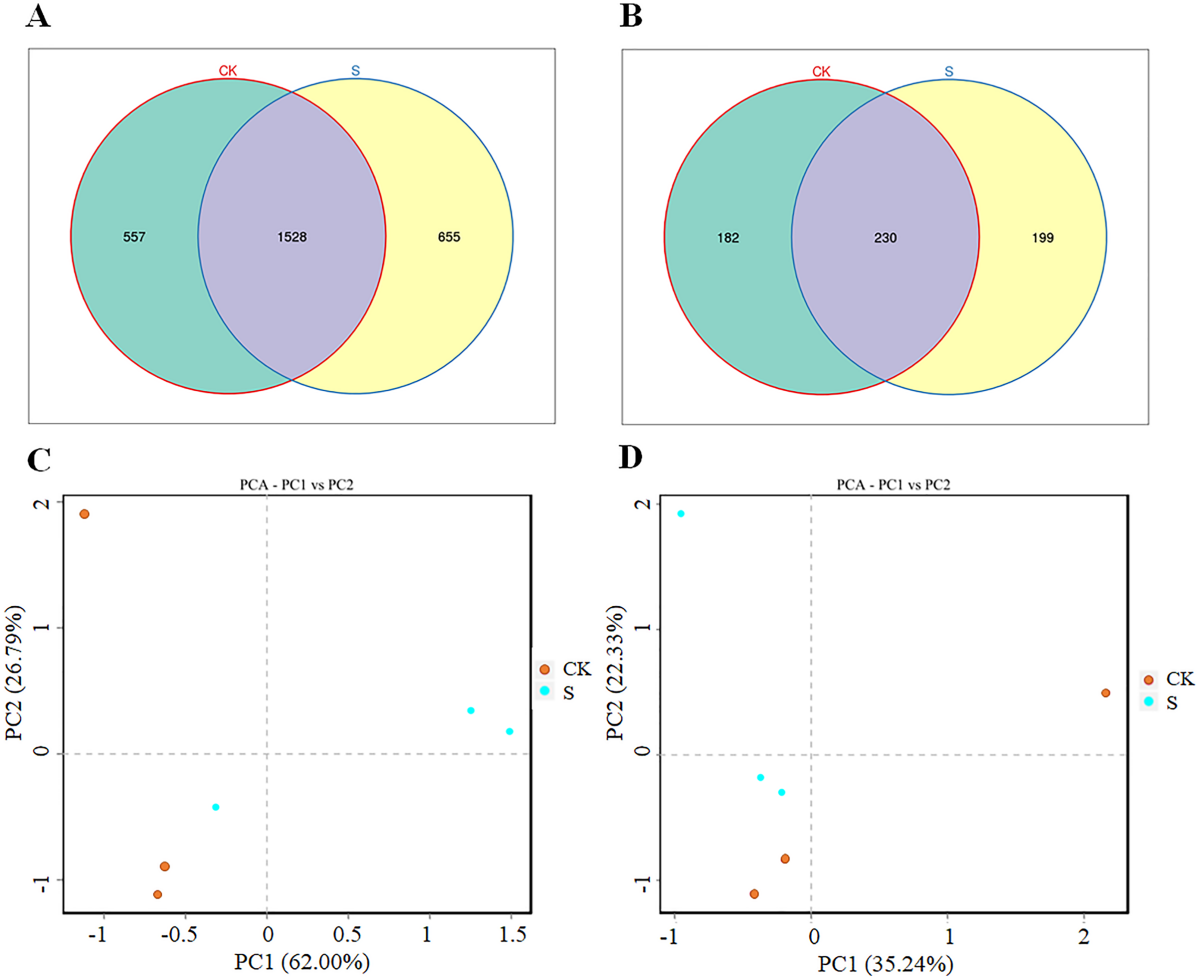
Rhizosphere microbial changes after yellowhorn growth in fly ash. (A) Venn diagrams of OTU number of bacteria, (B) venn diagrams of OTU number of fungi, (C) principal component analysis (PCA) of the rhizosphere bacterial community, (D) principal component analysis (PCA) of the rhizosphere fungal community.

Both the bacterial and fungal diversity increased significantly (*p* < 0.05) after yellowhorn cultivation (S group) compared with the pre-planting sample (CK group; Figs. 2A, 2B). There were 2,085 bacterial OTUs in the CK group and 2,193 OTUs in the S group with 1,528 OTUs overlapping in the two groups, 557 unique to the CK group, and 655 unique to the S group (Fig. 2A). The bacterial OTUs derived from 37 phyla and 896 genera (Table S3). There were 412 fungal OTUs in the CK group and 429 in the S group with 230 overlapping in the two groups, 182 unique to the CK group, and 199 unique to the S group (Fig. 2B). The fungal OTUs derived from 13 phyla and 170 genera (Table S4). The ANOSIM analysis found significant differences in both bacterial and fungal diversity between the S and CK groups (Figs. S2A, S2B). A PCA analysis showed that the rhizosphere bacterial (Fig. 2C) and fungal (Fig. 2D) communities in the fly ash after yellowhorn cultivation was significantly different from those in the CK group. These results indicate that the diversity of the rhizosphere microbial community increased in fly ash after planting yellowhorn.

### Rhizosphere microbial diversity after yellowhorn cultivation

**Figure 3:**
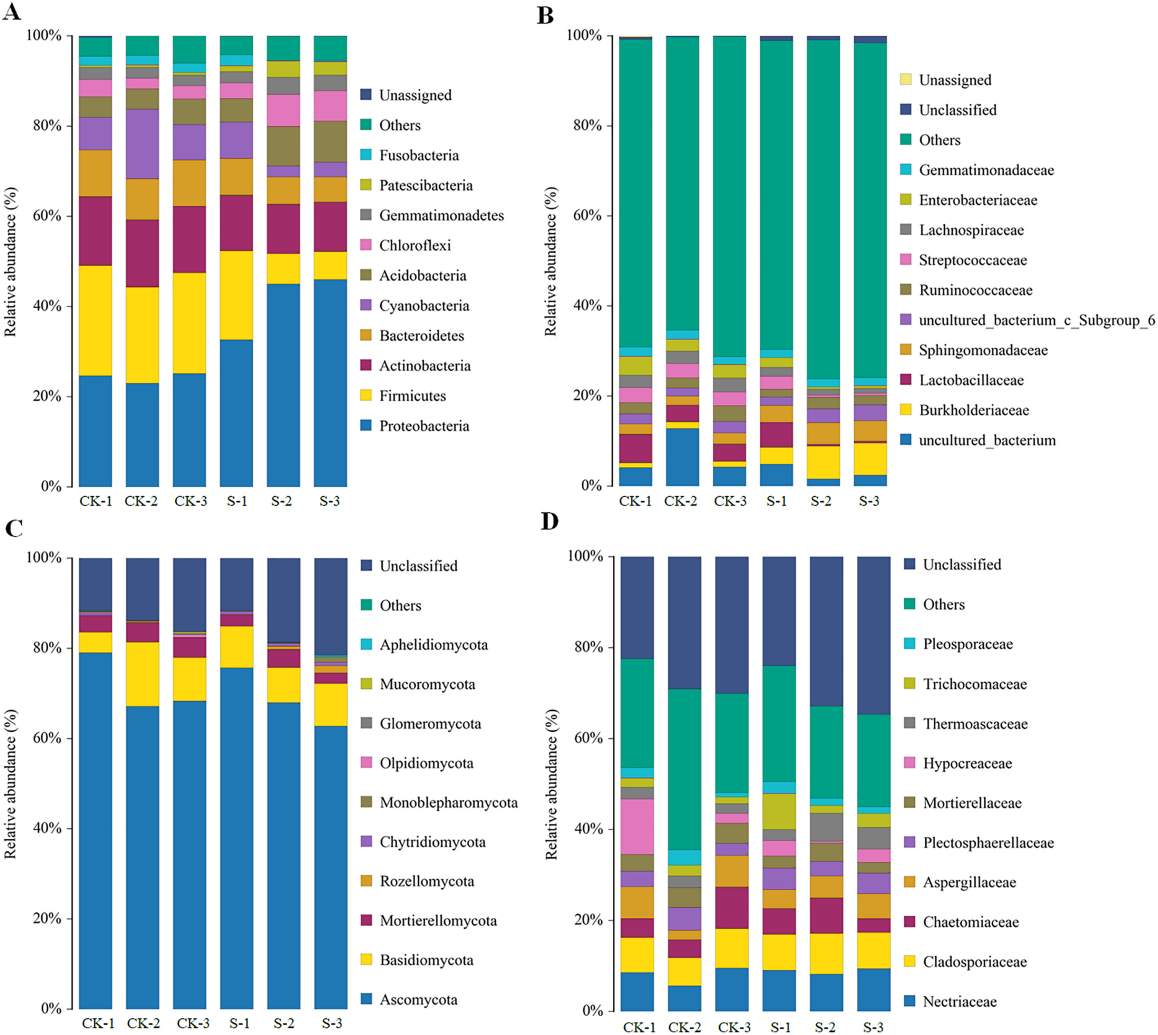
Changes in rhizosphere microbial community diversity at the sapling stages of yellowhorn cultivation. Relative abundances (%) of major bacterial diversity at the phylum (A) and family (B) levels. Relative abundances (%) of major fungal diversity at the phylum (C) and family (D) levels.

The majority of the bacterial OTUs could be assigned to ten major phyla (Fig. 3A), with eight of those 10 phyla: *Proteobacteria*, *Firmicutes*, *Actinobacteria*, *Bacteroidetes*, *Cyanobacteria*, *Acidobacteria*, *Chloroflexi*, and *Gemmatimonadetes* accounting for more than 90% of the total bacterial OTUs (Fig. 3A). Of these eight phyla, *Proteobacteria*, *Firmicutes*, *Bacteroidetes*, *Cyanobacteria*, *Acidobacteria*, and *Chloroflexi* changed dramatically with yellowhorn growth. At the sapling stage, the proportions of *Firmicutes*, *Actinobacteria*, *Bacteroidetes*, and *Cyanobacteria* were 11.0%, 11.3%, 6.7% and 4.3%, respectively, which were significantly lower (*p* < 0.01) than in the pre-planting stage, when their levels were 22.3%, 15.0%, 9.7% and 10.0%, respectively. However, the levels of *Proteobacteria*, *Acidobacteria*, *Chloroflexi*, and *Gemmatimonadetes* in the S group were 41.3%, 7.7%, 5.7% and 3.3%, respectively, which were significantly higher (*p* < 0.05) than in the pre-planting sample (24.3%, 5.3%, 3.0% and 2.3%, respectively; Fig. 3A). At the family level of bacterial OTUs, differences between the two groups were mainly caused by changes in the levels of *uncultured*_*bacterium*, *Burkholderiaceae*, *Sphingomonadaceae*, and *Streptococcaceae* (Fig. 3B).

The rhizosphere fungal community also mainly consisted of 10 dominant phyla with five of those 10 phyla: *Ascomycota*, *Basidiomycota*, *Mortierellomycota*, *Rozellomycota*, and *Chytridiomycota*, accounting for more than 80% of the fungal OTUs (Fig. 3C). *Ascomycota*, *Mortierellomycota* and *Chytridiomycota* levels were significantly different at the sapling stage, while other fungal phyla did not change significantly. At the sapling stage, *Ascomycota* and *Mortierellomycota* levels were 69.0% and 2.7%, respectively, which were significantly lower (*p* < 0.05) than at the pre-planting stage (71.3% and 4.0%, respectively), while *Chytridiomycota* levels were 0.70% at the sapling stage, which was significantly higher (*p* < 0.05) than at the pre-planting stage (0.20%) (Fig. 3C). At the family level, changes in the diversity of fungal OTUs were mainly caused by changes in the levels of *Chaetomiaceae*, *Thermoascaceae*, *Trichocomaceae*, and *Pleosporaceae* (Fig. 3D).

### Co-occurrence network, heatmap and phylogenetic analyses of the microbiomes

**Figure 4:**
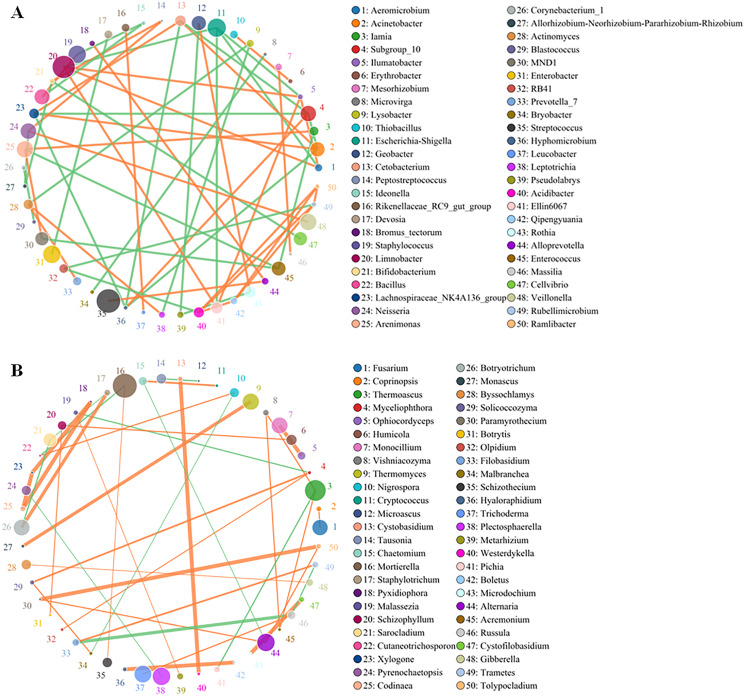
Co-occurrence network between the top 50 OTUs of absolute abundance of bacteria (A) and fungi (B). The size of the dot represents the abundance, and the thickness of the line represents the correlation; the color of the dot represents the genus, the orange line indicates a positive correlation, and the green line represents a negative correlation.

Although the yellowhorn microbiome changed significantly at the sapling stage, a core group of fungi and bacteria was present during all the growth stages of yellowhorn. The bacterial network was evenly divided between being positively and negatively correlated with the fungal network with an equal number of bacteria in each category (Fig. 4A). The bacterial community was dominated by six major genera: Escherichia-*Shigella*, *Staphylococcus*, *Limnobacter*, *Enterobacter*, *Streptococcus* and *Veillonella* (Fig. 4A) with most positively correlated with the fungal network (Fig. 4B). The fungal community was dominated by five major groups: *Thermoascus*, *Mortierella*, *Tricharina*, *Plectosphaerella* and *Alternaria* (Fig. 4B).

At the phylum level, 37 of the most abundant bacteria and 13 dominant fungi were analyzed using a heatmap between the CK and S groups. A total of 14 bacterial phyla had higher levels in the S group: *Elusimicrobia*, *Omnitrophicaeota*, *Chlproflexi*, *Gemmatimonadetes*, *Dependentiae*, *Acidobacteria*, *Nitrospirae*, *Proteobacteria*, *Patescibacteria*, FCPU426, *Fibrobacteres*, *Verrucomicrobia*, *Armatimonadetes*, and *WPS*-2 (Fig. S3A), and 12 phyla had higher levels in the CK group: *Planctomycetes*, *Rokubacterta*, *Thaumarchaeota*, *Entotheonellaeota*, *Synergistetes*, *Kiritimatiellaeota*, *Fusobacteria*, *Firmicutes*, *Epsilonbacteraeota*, *Spirochaetes*, *Actinobacteria*, and *Bacteroidetes* (Fig. S3A). Compared to the bacterial community, the fungal community showed different changes at the phylum level. Within the fungal community, both *Chytridiomycota* and *Rozellomycota* were increased in the S group (Fig. S3B).

The phylogenetic trees of both bacterial and fungal OTUs were constructed at the genus level (Fig. S4). In the phylogenetic tree of bacterial OTUs (Fig. S4A), 27 OTU sequences with the highest abundance belonged to *Proteobacteria*, and 16 OTU sequences with the highest abundance belonged to *Actinobacteria*. In the phylogenetic tree of fungal OTUs (Fig. S4B), 51 genus OTU sequences with the highest abundance belonged to *Ascomycota*, and 26 genus OTU sequences with the highest abundance belonged to *Basidiomycota*.

### Differences in rhizosphere microbial community diversity between the sapling and pre-planting stages

Alpha-biodiversity reflects species abundance and diversity (Ding et al., 2020; Lin et al., 2019). In this study, the Chao1, Ace (Abundance-based Coverage Estimator), Shannon, and Simpson indices were used to measure Alpha diversity and the Chao1 and Ace indices were used to measure richness. The Chao1 and Ace indices of the S group were significantly lower in bacterial richness than those of the CK group, indicating that the abundance of bacteria in fly ash decreased slightly at the sapling stage of yellowhorn (Table 1). The abundance of fungi in fly ash also decreased slightly after planting yellowhorn according to the Chao1 and Ace indices for fungal richness (Table 1). The Shannon and Simpson indices were used to measure species diversity (Lin et al., 2021). However, neither the Simpson or Shannon index of bacterial and fungal diversity changed significantly between the sapling and pre-planting stages (Table 1).

**Table 1:** Alpha-biodiversity indices of bacteria and fungi in fly ash between the sapling and pre-planting stages of yellowhorn.

Classification	Sample ID	Ace	Chao1	Simpson	Shannon
Bacteria	CK	1,517.6 ± 21.9	1,535.2 ± 22.6	0.0075 ± 0.0040	6.2 ± 0.1
	S	1,445.9 ± 25.4*	1,508.9 ± 28.2*	0.0050 ± 0.0001	6.3 ± 0.1
Fungi	CK	244.2 ± 24.8	245.6 ± 27.9	0.0212 ± 0.0056	4.5 ± 0.1
	S	222.3 ± 36.8*	214.1 ± 41.4*	0.0200 ± 0.0006	4.5 ± 0.2

**Note:**

* represent the significant difference at the 0.05 level.

**Figure 5:**
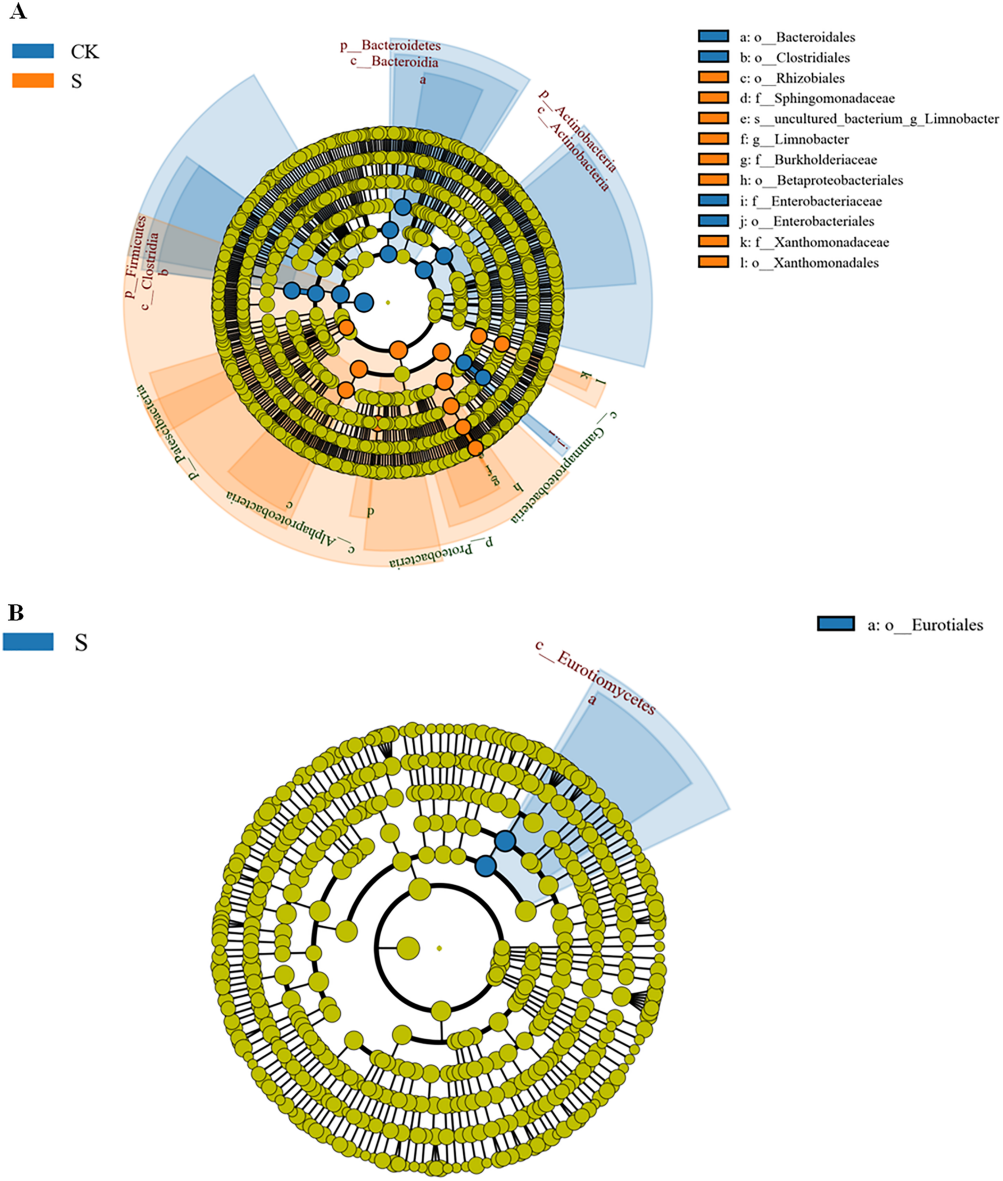
LDA effect size phylogenetic cladogram of bacteria (A) and fungal (B) LEfSe between the sapling and pre-planting stages. The phylum (p), class (c), order (o), family (f), genus (g) names indicated by the letters in the figure are shown in the legend on the right. The circle radiating from the inside to the outside of the branch diagram represents the classification level from phylum to genus (or species). Each small circle at a different classification level represents a classification at that level, and the diameter of the circle corresponds to the relative abundance. Different colors indicate different groups, and nodes of different colors indicate the groups of microorganisms that play an important role in the groups represented by the colors.

As stated above, there were strong differences in rhizosphere microbial community richness at the sapling stage. A LEfSe (Line Discriminant Analysis (LDA) Effect Size) analysis was used to identify representative microbes (Segata et al., 2011) using an LDA score >4 (Figs. S5A and S5B). The Lefse evolutionary branching diagrams of bacteria and fungi in fly ash samples between the CK and S groups are shown in Fig. 5. At the bacterial taxonomic level, *o-Rhizobiales, f-Sphingomonadaceae, s-unculted-bacterium-g-Limnobacter, g-Limnobacter, f-Burkholderiacae, o-Betaproteobacteriales, f-Xanthomonadaceae*, and *o-Xanthomonadales* were significantly enriched at the sapling stage (Fig. 5A), most of which could be used for phytoremediation. At the fungal order level, the abundance of *o-Eurotiales* was significantly enriched at the sapling stage (Fig. 5B).

### Heavy metal content changes in rhizosphere fly ash and different tissues of yellowhorn

**Figure 6:**
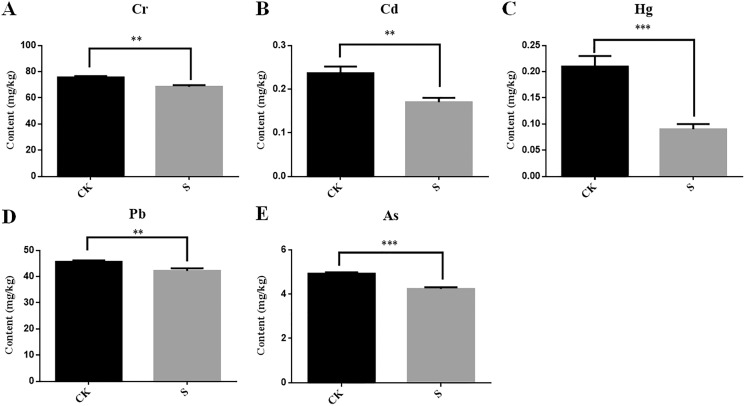
Determination of the content of five heavy metals Cr (A), Cd (B), Hg (C), Pb (D) and As (E) in fly ash between the sapling and pre-planting stages. Asterisks (** and ***) represent the significant difference at the 0.01 and 0.001 levels, respectively.

During yellowhorn plant growth, we found that the content of five heavy metals (Cr, Cd, Hg, Pb and As) in rhizosphere fly ash decreased at the sapling stage (Figs. 6A–6E). Among them, Cd and Pb could be detected in the fibrous roots, taproots, stems and leaves of the yellowhorn plants, and Cr and As were mainly detected in the fibrous roots, taproots, and stems of the yellowhorn plants (Table 2). These results indicate that yellowhorn can absorb Cd, Pb, Cr and As through its root system and transport them to other tissues, such as stems and leaves. In addition, Hg decreased significantly in the S group, but it was not detected in the roots, stems, or leaves of yellowhorn plants, which might indicate that microorganism activity caused Hg levels to decrease rather than being absorbed by the yellowhorn like the other metals. These results show that the physicochemical properties of fly ash might be related to rhizosphere microbial community diversity and that planting yellowhorn for phytoremediation could reduce heavy metal content in fly ash.

**Table 2:** Heavy metal contents of different yellowhorn tissues cultivated in the fly ash.

Tissues	Cr (mg/kg)	Cd (mg/kg)	Hg (mg/kg)	Pb (mg/kg)	As (mg/kg)
Taproot	4.35 ± 0.21	0.14 ± 0.05	ND	0.55 ± 0.12	0.06 ± 0.01
Fibrous root	15.67 ± 0.28	0.38 ± 0.05	ND	3.36 ± 0.19	0.50 ± 0.22
Stem	9.86 ± 013	0.10 ± 0.03	ND	0.30 ± 0.08	ND
Leaf	ND	0.10 ± 0.02	ND	0.10 ± 0.03	ND

**Note:**

ND, Not detected.

## Discussion

Plant growth can change a number of soil environmental factors and affect the composition and function of the microbial community in the soil (Kavamura et al., 2013; Kodobocz & Muranyi, 2008; Na et al., 2018). Bang-Andreasen et al. (2020) found that wood ash application affected the abundance and diversity of bacteria in both agricultural and forest soil more than fungal abundance and diversity. In our study, planting yellowhorn changed the diversity of both bacteria and fungi in fly ash. The dominant phylum in the healthy soil bacterial library are: *Proteobacteria, Acidobacteria, Actinobacteria, Verrucomicrobia, Bacteroidetes, Chloroflexi, Planctomycetes, Gemmatimonadetes*, and *Firmicutes* (Janssen, 2006). In our fly ash samples, the second dominant phyla, *Firmicutes*, decreased in proportion, while *Proteobacteria* and *Acidobacteria* increased, showing that plant growth promotes the rapid transformation of the bacterial community to primarily *Proteobacteria* and *Acidobacteria* (Singh et al., 2007). *Proteobacteria* and *Acidobacteria* belong to gram-negative bacteria, which are more resistant to metal contamination than gram-positive bacteria (Fei et al., 2010).

The heavy metal content of fly ash can stress the growth of microorganisms, but planting yellowhorn increased microbial diversity, which helped the absorption and degradation of the heavy metal content in the fly ash. In our study of the microbial community of the fly ash at the sapling stage of yellowhorn, we found that *Proteobacteria* significantly increased in the S group compared with the CK group. *Proteobacteria* can solubilize heavy metals so they can be easily absorbed by plants, allowing the plants to remove heavy metals from the fly ash through absorption (Lin et al., 2021). In addition, *s-uncultured-bacterium-g-limnobacter* is a thiosulfate oxidizing bacterium, and the increased content of limnobacter we observed in this study indicates that rhizosphere fly ash is more acidic (Spring, Kampfer & Schleifer, 2001). Soil acidification makes it easier for heavy metals to enter the soil solution and migrate to plant roots, where they can be absorbed by plants (Blake & Goulding, 2002; Yang et al., 2010), further promoting the degradation of heavy metals by *Proteobacteria*. The enriched bacteria, *Gammaproteobacteria*, have been repeatedly found in nutrient-rich sites such as the rhizosphere, which might imply that the fly ash is nutrient-rich after planting yellowhorn (Ernebjerg & Kishony, 2012; Fierer et al., 2012).

The fertility of the fly ash also changed after yellowhorn planting. For instance, the abundance of *Acidobacteria* in the microbial community increased in the fly ash at the yellowhorn sapling stage. *Acidobacteria* participates in the carbon cycle and degrades plant polysaccharides, such as cellulose and lignin (Hou et al., 2020). In addition, *Nitrospirae*, *Betaproteobacteriales* and *Burkholderiacae*, which participate in nitrite oxidation and nitrogen fixation, and provide nitrogen to plant roots to promote plant growth (Boddey et al., 2003; Chiarini et al., 1998; Koch et al., 2015; Lücker et al., 2010; Van et al., 2000), also increased. *Rhizobiales*, a bacterium that is beneficial to plant symbiosis for the remediation of heavy metals in soil, is the predominant bacterial OTU in fly ash samples with yellowhorn plants (Ren et al., 2019). *Sphingomonadaceae* absorbs heavy metal Cd and its high abundance in this study with yellowhorn shows great potential for environmental protection (Baraniecki, Aislabie & Foght, 2002). The roots of different plants form their own microregions, which make the microbial community structure of rhizosphere soil conducive to soil remediation (Lin et al., 2021). The diversity of microorganisms both directly and indirectly help the yellowhorn plants absorb the heavy metals in fly ash, and thus contribute to the phytoremediation of the fly ash.

In our study, we investigated the microbial diversity of fly ash after yellowhorn cultivation. The relative abundance of nitrogen-fixing bacteria in the rhizosphere microorganisms of yellowhorn increased, benefiting the growth of the yellowhorn in the fly ash (Rau et al., 2009). The benefits of fly ash bioremediation through yellowhorn planting were significant: the heavy metal content of the fly ash was reduced with the help of rhizosphere microorganisms that produced acidic organic matter which helped the root systems of the plants absorb the heavy metals (Delvasto et al., 2009; Han et al., 2006). The seeds of yellowhorn can also be used as biofuel. The results of our study indicate that the bioremediation of fly ash through the cultivation of yellowhorn should be the topic of subsequent research.

## Conclusions

This article compared the microbial diversity of fly ash in the sapling stage of yellowhorn cultivation to the pre-planting control. The results showed that planting yellowhorn changed the abundance and diversity of bacteria and fungi in the rhizosphere fly ash. In addition, planting yellowhorn had a larger effect on bacteria than on fungi. The proportion of *Proteobacteria* and *Acidobacteria* in the bacterial community, which are both conducive to soil carbon cycling, increased significantly. The abundance of bacteria whose functions are closely related to the environmental cycling of heavy metals, including *Nitrospirae, Betaproteobacteriales, Burkholderiacae, Rhizobiales*, and *Sphingomonadaceae*, also increased. The heavy metal content of the fly ash and the absorption of some of those heavy metals by the yellowhorn plants also affected the microbial diversity of the rhizosphere. Although our study did not elucidate the interaction mechanisms between the rhizosphere microbiome and yellowhorn plants, our results provide important clues for understanding the dynamic changes of microbial community diversity in the rhizosphere during yellowhorn cultivation, which would be useful for the phytoremediation of fly ash.

## Supplemental Information

10.7717/peerj.14015/supp-1 Supplemental Information 1 Sequencing statistics and basic OTU analyses. (A) bacterial 16S DNA Shannon-Wiener curve, (B) fungal ITS Shannon-Wiener curve, (C) bacterial 16S DNA Rarefaction curve, (D) fungal ITS Rarefaction curve, (E) bacterial species OTUs number, (F) fungal species OTUs number.

10.7717/peerj.14015/supp-2 Supplemental Information 2 ANOSIM analysis boxplot. The R-value (between -1 and 1) is greater than 0, indicating that the difference between the groups is significant. The credibility of the statistical analysis is expressed by P-value, and P <0.05 indicates significance.

10.7717/peerj.14015/supp-3 Supplemental Information 3 Heatmap analysis of the abundance at the bacterial (A) and fungal (B) phylum levels. Heatmap analysis of the abundance at the bacterial (A) and fungal (B) phylum levels.

10.7717/peerj.14015/supp-4 Supplemental Information 4 Phylogenetic tree of OTU of bacteria (A) and fungi (B) at the genus level. In the phylogenetic tree, the ring diagram showed the species evolution tree, and the genus with the same color belonged to the same phylum.

10.7717/peerj.14015/supp-5 Supplemental Information 5 Distribution histogram of LDA effect size analysis based on classification information. The figure shows microorganisms with significantly different abundances between CK and S groups with LDA score greater than 4. The length of the histogram represents the size of the influence of the significantly different microorganisms.

10.7717/peerj.14015/supp-6 Supplemental Information 6 Statistics of bacteria 16S DNA sequencing data.

10.7717/peerj.14015/supp-7 Supplemental Information 7 Statistics of fungal ITS sequencing data.

10.7717/peerj.14015/supp-8 Supplemental Information 8 Statistics of the bacterial OTUs number at different taxonomy levels: kingdom, phylum, class, order, family, genus and species.

10.7717/peerj.14015/supp-9 Supplemental Information 9 Statistics of the fungal OTUs number at different taxonomy levels: kingdom, phylum, class, order, family, genus and species.

10.7717/peerj.14015/supp-10 Supplemental Information 10 The raw data for Figure 6.

10.7717/peerj.14015/supp-11 Supplemental Information 11 The raw data for Table 2.

## Additional Information and Declarations

### Data Availability

The following information was supplied regarding data availability: The sequencing raw data are available at the Genome Sequence Archive: CRA005791.
