# Supplementary figures and images for: Influence of planting yellowhorn (Xanthoceras sorbifolium Bunge) on the bacterial and fungal diversity of fly ash
Source: PeerJ. 2022 Sep 23;10:e14015. doi: 10.7717/peerj.14015 (PMC9512002; doi:10.7717/peerj.14015)

**A**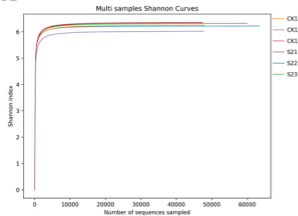**B**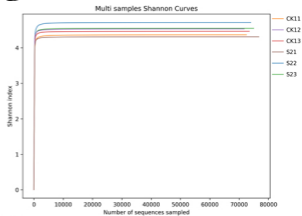**C**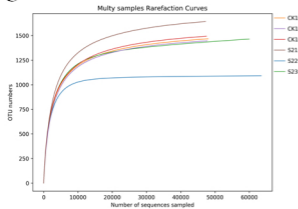**D**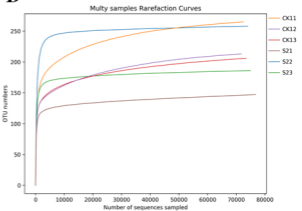**E**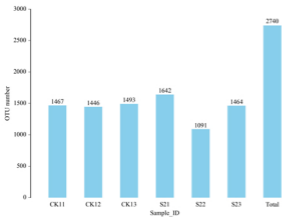**F**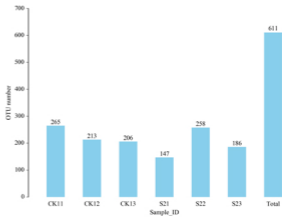

Supplement: Supplemental Information 1 — (A) bacterial 16S DNA Shannon-Wiener curve, (B) fungal ITS Shannon-Wiener curve, (C) bacterial 16S DNA Rarefaction curve, (D) fungal ITS Rarefaction curve, (E) bacterial species OTUs number, (F) fungal species OTUs number. [file peerj-10-14015-s001.pdf]

**A**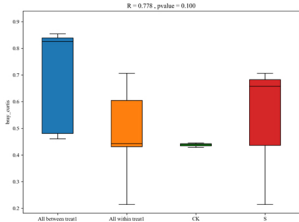**B**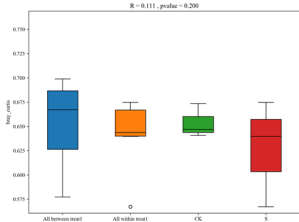

Supplement: Supplemental Information 2 — The R-value (between -1 and 1) is greater than 0, indicating that the difference between the groups is significant. The credibility of the statistical analysis is expressed by P-value, and P <0.05 indicates significance. [file peerj-10-14015-s002.pdf]

A

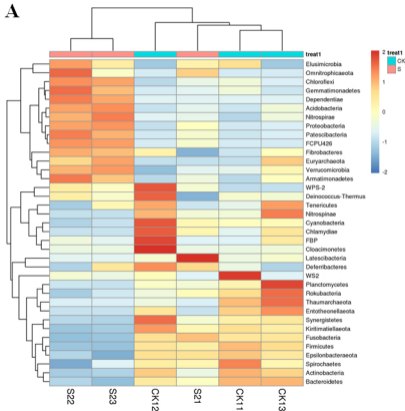

B

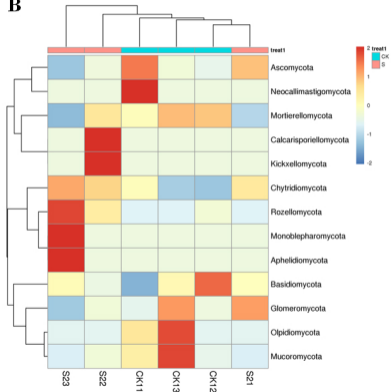

Supplement: Supplemental Information 3 — Heatmap analysis of the abundance at the bacterial (A) and fungal (B) phylum levels. [file peerj-10-14015-s003.pdf]

A

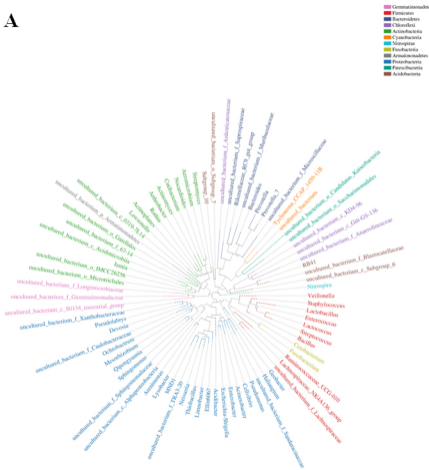

B

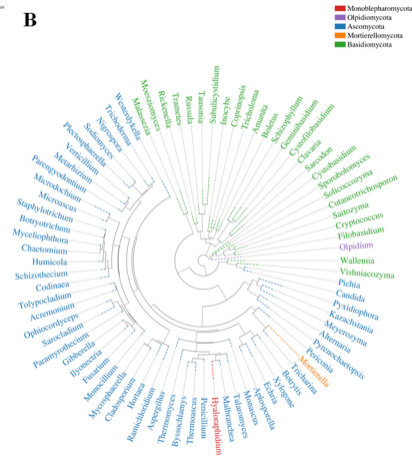

Supplement: Supplemental Information 4 — In the phylogenetic tree, the ring diagram showed the species evolution tree, and the genus with the same color belonged to the same phylum. [file peerj-10-14015-s004.pdf]

**A**

CK

S

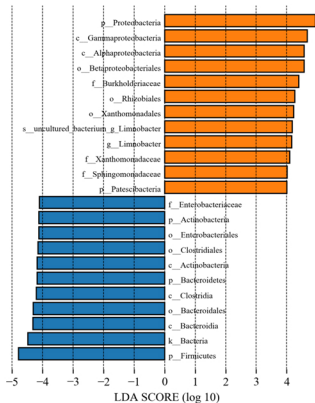**B**

S

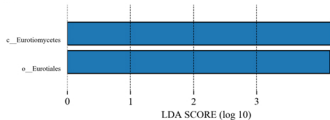

Supplement: Supplemental Information 5 — The figure shows microorganisms with significantly different abundances between CK and S groups with LDA score greater than 4. The length of the histogram represents the size of the influence of the significantly different microorganisms. [file peerj-10-14015-s005.pdf]
