# Supplementary material for: Influence of planting yellowhorn (Xanthoceras sorbifolium Bunge) on the bacterial and fungal diversity of fly ash
Source: PeerJ. 2022 Sep 23;10:e14015. doi: 10.7717/peerj.14015 (PMC9512002; doi:10.7717/peerj.14015)
Supplement: Supplemental Information 6 [file peerj-10-14015-s006.docx]

**S1 Table. Statistics of bacteria 16S DNA sequencing data.**

| Sample | PE Reads | Raw Tags | Clean Tags | Effective Tags | AvgLen(bp) | GC(%) | Q20(%) | Q30(%) | Effective(%) |
| --- | --- | --- | --- | --- | --- | --- | --- | --- | --- |
| CK-1 | 79,780 | 78,926 | 77,316 | 71,531 | 419 | 54.94 | 97.95 | 95.75 | 89.66 |
| CK-2 | 80,015 | 79,094 | 77,475 | 71,796 | 417 | 54.94 | 97.93 | 95.71 | 89.73 |
| CK-3 | 79,647 | 78,716 | 77,049 | 70,694 | 418 | 55.08 | 97.92 | 95.69 | 88.76 |
| S-1 | 79,587 | 78,631 | 77,059 | 72,046 | 418 | 54.91 | 97.89 | 95.65 | 90.52 |
| S-2 | 79,820 | 79,216 | 77,611 | 76,432 | 417 | 55.47 | 97.87 | 95.62 | 95.76 |
| S-3 | 79,710 | 79,070 | 77,503 | 76,149 | 417 | 55.49 | 97.93 | 95.73 | 95.53 |
