# Supplementary material for: Influence of planting yellowhorn (Xanthoceras sorbifolium Bunge) on the bacterial and fungal diversity of fly ash
Source: PeerJ. 2022 Sep 23;10:e14015. doi: 10.7717/peerj.14015 (PMC9512002; doi:10.7717/peerj.14015)
Supplement: Supplemental Information 7 [file peerj-10-14015-s007.docx]

**S2 Table.** Statistics of fungal ITS sequencing data

| Sample | PE Reads | Raw Tags | Clean Tags | Effective Tags | AvgLen(bp) | GC(%) | Q20(%) | Q30(%) | Effective(%) |
| --- | --- | --- | --- | --- | --- | --- | --- | --- | --- |
| CK-1 | 80,080 | 74,867 | 74,866 | 73,373 | 234 | 50.40 | 99.87 | 99.56 | 91.62 |
| CK-2 | 80,083 | 72,409 | 72,409 | 72,376 | 235 | 48.60 | 99.85 | 99.53 | 90.38 |
| CK-3 | 79,817 | 75,031 | 75,031 | 74,330 | 237 | 49.00 | 99.86 | 99.53 | 93.13 |
| S-1 | 79,893 | 77,103 | 77,101 | 77,066 | 243 | 49.69 | 99.84 | 99.51 | 96.46 |
| S-2 | 79,985 | 74,705 | 74,704 | 74,623 | 242 | 50.32 | 99.84 | 99.49 | 93.30 |
| S-3 | 80,101 | 75,585 | 75,585 | 75,289 | 244 | 49.40 | 99.83 | 99.49 | 93.99 |
