# Supplementary material for: Influence of planting yellowhorn (Xanthoceras sorbifolium Bunge) on the bacterial and fungal diversity of fly ash
Source: PeerJ. 2022 Sep 23;10:e14015. doi: 10.7717/peerj.14015 (PMC9512002; doi:10.7717/peerj.14015)
Supplement: Supplemental Information 8 [file peerj-10-14015-s008.docx]

**S3 Table.** Statistics of the bacterial OTUs number at different taxonomy levels: kingdom, phylum, class, order, family, genus and species.

| **Sample** | **Kingdom** | **Phylum** | **Class** | **Order** | **Family** | **Genus** | **Species** |
| --- | --- | --- | --- | --- | --- | --- | --- |
| CK-1 | 2 | 31 | 72 | 172 | 305 | 617 | 678 |
| CK-2 | 2 | 32 | 78 | 185 | 317 | 640 | 693 |
| CK-3 | 2 | 31 | 76 | 182 | 313 | 638 | 693 |
| S-1 | 1 | 30 | 80 | 190 | 333 | 690 | 751 |
| S-2 | 2 | 28 | 75 | 177 | 298 | 489 | 519 |
| S-3 | 2 | 31 | 82 | 198 | 336 | 615 | 666 |
| Total | 2 | 37 | 99 | 251 | 433 | 896 | 995 |
