# Supplementary material for: Influence of planting yellowhorn (Xanthoceras sorbifolium Bunge) on the bacterial and fungal diversity of fly ash
Source: PeerJ. 2022 Sep 23;10:e14015. doi: 10.7717/peerj.14015 (PMC9512002; doi:10.7717/peerj.14015)
Supplement: Supplemental Information 9 [file peerj-10-14015-s009.docx]

**S4 Table.** Statistics of the fungal OTUs number at different taxonomy levels: kingdom, phylum, class, order, family, genus and species.

| **Sample** | **Kingdom** | **Phylum** | **Class** | **Order** | **Family** | **Genus** | **Species** |
| --- | --- | --- | --- | --- | --- | --- | --- |
| CK11 | 1 | 8 | 21 | 44 | 78 | 96 | 90 |
| CK12 | 1 | 7 | 18 | 42 | 74 | 86 | 75 |
| CK13 | 1 | 8 | 19 | 41 | 62 | 73 | 63 |
| S21 | 1 | 5 | 16 | 31 | 54 | 67 | 53 |
| S22 | 1 | 10 | 22 | 44 | 74 | 91 | 75 |
| S23 | 1 | 7 | 18 | 36 | 57 | 67 | 56 |
| Total | 1 | 13 | 31 | 65 | 126 | 170 | 159 |
